# Glutamatergic and dopaminergic function and the relationship to outcome in people at clinical high risk of psychosis: a multi-modal PET-magnetic resonance brain imaging study

**DOI:** 10.1038/s41386-019-0541-2

**Published:** 2019-10-16

**Authors:** Oliver D. Howes, Ilaria Bonoldi, Robert A. McCutcheon, Matilda Azis, Mathilde Antoniades, Matthijs Bossong, Gemma Modinos, Jesus Perez, James M. Stone, Barbara Santangelo, Mattia Veronese, Anthony Grace, Paul Allen, Philip K. McGuire

**Affiliations:** 1 Institute of Psychiatry, Psychology and Neuroscience, King’s College London, De Crespigny Park, Camberwell, London, SE5 8AF UK; 2 MRC London Institute of Medical Sciences, Imperial College London, Hammersmith Hospital, Du Cane Road, London, W12 0NN UK; 3 TREAT Service, South London and Maudsley Foundation NHS Trust, Maudsley Hospital, London, SE5 8AZ UK; 4 Department of Psychiatry, University Medical Center Utrecht, Utrecht, The Netherlands; 5 Cambridge Early Onset service, Cambridgeshire and Peterborough Mental Health Partnership National Health Service Trust, Cambridge, UK; 6 Department of Psychiatry, University of Cambridge, Cambridge, UK; 7 Department of Neuroscience, Psychiatry and Psychology, University of Pittsburgh, Pittsburgh, PA USA; 8 Department of Psychology, University of Roehampton, London, UK

**Keywords:** Predictive markers, Perception

## Abstract

Preclinical models of psychosis propose that hippocampal glutamatergic neuron hyperactivity drives increased striatal dopaminergic activity, which underlies the development of psychotic symptoms. The aim of this study was to examine the relationship between hippocampal glutamate and subcortical dopaminergic function in people at clinical high risk for psychosis, and to assess the association with the development of psychotic symptoms. ^1^H-MRS was used to measure hippocampal glutamate concentrations, and ^18^F-DOPA PET was used to measure dopamine synthesis capacity in 70 subjects (51 people at clinical high risk for psychosis and 19 healthy controls). Clinical assessments were undertaken at baseline and follow-up (median 15 months). Striatal dopamine synthesis capacity predicted the worsening of psychotic symptoms at follow-up (*r* = 0.35; *p* < 0.05), but not transition to a psychotic disorder (p = 0.22), and was not significantly related to hippocampal glutamate concentration (*p* = 0.13). There were no differences in either glutamate (*p* = 0.5) or dopamine (*p* = 0.5) measures in the total patient group relative to controls. Striatal dopamine synthesis capacity at presentation predicts the subsequent worsening of sub-clinical total and psychotic symptoms, consistent with a role for dopamine in the development of psychotic symptoms, but is not strongly linked to hippocampal glutamate concentrations.

## Introduction

Psychotic disorders are typically preceded by a prodromal period of 1–5 years, characterised by worsening sub-clinical psychotic symptoms and a decline in overall functioning [1]. Operationalised criteria have been developed on the basis of symptoms, functional decline and family history of psychosis to identify people who may be in the prodrome and are at high risk of developing psychosis in the next few years [2]. People meeting these operationalised criteria are referred to as being at clinical high risk of psychosis. Psychotic disorders are associated with considerable burden once they have developed, and current treatments are limited by poor tolerability and effectiveness in many patients [3]. There is thus considerable interest in understanding the pathophysiology underlying the development of psychotic symptoms to help develop new treatments [4].

The dopamine and glutamate hypotheses are two leading theories for the pathophysiology of psychosis [3]. Although initially developed separately, the hypotheses have been integrated to propose that glutamate dysregulation in cortical regions, including the hippocampus, leads to striatal dopamine dysfunction, which, in turn, underlies the development of psychotic symptoms [3, 5–9]. Supporting this, preclinical evidence indicates that hippocampal hyperactivity leads to mesostriatal dopamine dysfunction [10–12], and implicates glutamatergic neuron dysregulation in the hippocampus in this process [13]. Moreover, a number of clinical studies have shown altered hippocampal structure, function and perfusion in psychotic disorders, and people at risk of psychosis [13–20]. However, to our knowledge, only one study has investigated the relationship between hippocampal glutamatergic measures and striatal dopamine function in people at high risk for psychosis [21]. This study found levels of glutamate, in the hippocampus, were inversely related to striatal dopamine synthesis capacity in people at clinical risk of psychosis, particularly in the subgroup who went on to develop a psychotic disorder. This suggests the hypothesis that dysfunction in hippocampal glutamatergic drive dysregulates striatal dopaminergic function. As the number of subjects in the one in vivo study in patients to date was relatively modest (*n* = 14), the first aim of this study was to test the hypothesis that striatal dopamine synthesis capacity and hippocampal glutamate measures are related in an independent and larger sample of clinical high-risk subjects.

In vivo imaging studies of dopaminergic function show that dopamine synthesis and release capacity are elevated in patients with psychosis [22, 23], and some [24, 25], but not all [26, 27], studies have found this to be associated with the severity of psychotic symptoms. Striatal dopamine-release capacity has also been associated with the induction of psychotic symptoms by amphetamine [27]. Elevated dopamine synthesis and release capacity have also been reported in people at clinical high risk of psychosis [28–30], but not in other groups experiencing sub-threshold psychotic symptoms who do not meet criteria for a psychotic disorder [31]. Raised dopamine synthesis capacity has also been shown to be specific to those individuals who associated with an increased risk of transition to psychosis [32, 33]. Taken together, this evidence suggests that dopaminergic dysfunction may underlie the development of psychotic symptoms. However, it is not known if dopamine synthesis capacity predicts the worsening of sub-clinical psychotic symptoms in people at risk of psychosis. Therefore, the second aim of our study was to test the hypothesis that increased striatal dopamine synthesis capacity would predict an increase in the severity of psychotic symptoms, as well as the onset of a psychotic disorder.

## Methods

### Study design

The study comprised a case–control comparison of patients with healthy controls with a longitudinal, naturalistic clinical follow-up of patients to determine the relationship between baseline imaging measures and clinical outcome. All volunteers received clinical and imaging measures. In addition, the patients received follow-up to determine clinical outcome as described below. Ethical permission was given by the local research ethics committee. All participants provided written informed consent to participate.

### Participants

Patients were recruited from services for people at clinical high risk of psychosis in South England. Inclusion criteria were (1) met operationalized criteria for being at clinical high risk of psychosis based on a standardised, semi-structured clinical assessment as described by Yung et al. [34]; (2) no history of current or past psychotic disorder assessed using the structured clinical interview for diagnosis [35] and (3) antipsychotic naive or antipsychotic-free for at least 6 weeks.

Healthy controls were recruited from the same geographical area via adverts in the local media and met the following inclusion criteria: (1) no history of current or past mental disorder assessed using the structured clinical interview for diagnosis [35]; (2) no criteria for being at clinical high risk of psychosis as described by Yung et al. [34] (3) antipsychotic naive or antipsychotic-free.

Exclusion criteria for all subjects were any of (1) a history of significant head trauma, (2) dependence on illicit substances, (3) medical co-morbidity (other than minor, self-limiting illnesses) and (4) contraindications to scanning (such as pregnancy).

### Clinical measures

At baseline, all subjects were assessed using the Global Assessment of Function (GAF [36]) to measure social and occupational function, and the National Adult Reading Test (NART) to estimate premorbid IQ [37]. In addition, all patients received symptom measures rated using the Positive and Negative Syndrome Scale (PANSS [38]) and the Comprehensive Assessment of At Risk Mental States (CAARMS [34]) rating scales at baseline and at clinical follow-up.

### Clinical follow-up

Patients received a follow-up assessment at a median of 15.0 (interquartile range (IQR) = 11–23) months post baseline measures. Transition to a psychotic disorder was determined using the structured clinical interview for diagnosis [35]. The percentage change in CAARMS-positive symptom (unusual thought content, bizarre ideas, perceptual abnormalities and disorganised speech) severity rating from baseline to follow-up was calculated as follows:

$$	100 \times ({\mathrm{symptom}} {\mathrm{rating}} {\mathrm{at}} {\mathrm{follow}} {\mathrm{up}} - {\mathrm{symptom}} {\mathrm{rating}} {\mathrm{at}} {\mathrm{baseline}}){\mathrm{/}}\\ 	 ({\mathrm{symptom}} {\mathrm{rating}} {\mathrm{at}} {\mathrm{baseline}} )$$

where CAARMS scores were not available for patients (*n* = 3), PANSS-positive severity ratings (after subtracting seven to correct for the non-zero floor [39]) were used in their place to calculate the percentage change in positive symptoms.

### PET imaging acquisition and analysis

All participants received a PET scan to index dopamine synthesis capacity at baseline. Subjects were asked not to eat or drink (except water), and refrain from alcohol for 12 h prior to scan and not to smoke for 2 h prior to the scan [40]. Imaging data were obtained on a Siemens Biograph 6 HiRez PET scanner (Siemens, Erlangen, Germany) in three-dimensional mode. To prevent formation of radiolabelled metabolites that may cross the blood–brain barrier, participants received 400 mg of entacapone, a peripheral catechol-o-methyl-transferase inhibitor, and 150 mg of carbidopa, a peripheral aromatic acid decarboxylase inhibitor, 1 h before scan. Participants were positioned in the scanner with the orbitomeatal line parallel to the transaxial plane of the tomograph. Head position was marked and monitored, and a CT scan was conducted for attenuation correction. Approximately 150 MBq of ^18^F-DOPA was administered by bolus intravenous injection 30 s after the start of the dynamic PET scan. PET data were acquired in 32 frames of increasing duration, over the 95-min scan (frame intervals: 8 × 15, 3 × 60, 5 × 120, 16 × 300 s). Our primary measure was the whole striatal influx constant (K_i_^cer^, described as Ki in some earlier publications [28]).

Image analysis was conducted blind to group status. A mutual information algorithm was used to correct for head movement [41]. SPM8 was used to automatically normalise a tracer-specific ^18^F-DOPA template [42], together with a striatal brain atlas using the definition described by Martinez et al., which includes dividing the striatum into three subdivisions based on the predominant origin of projections to the striatum from limbic, associative and sensorimotor brain regions, respectively [43]. The whole striatum was our primary region of interest. However, given recent findings that the elevation in dopamine synthesis capacity in psychosis may be more marked in the associative striatum [23], we conducted additional exploratory analyses using the subdivisions for completeness. Following visual inspection of the time-activity curves, K_i_^cer^ was calculated using the Patlak–Gjedde graphical approach adapted for a reference tissue input function [44]. We have previously shown this approach to have good reliability, with intraclass correlation coefficients for the whole striatum of over 0.8 [45].

### MRS acquisition

Scanning was conducted on a General Electric (Milwaukee, Wisconsin, USA) Signa HDxt 3Tesla MRI scanner. Structural images were acquired using a whole-brain three-dimensional sagittal T1-weighted scan, with parameters based on the Alzheimer’s Disease Neuroimaging Initiative (ADNI) (TE = 2.85 ms; TR = 6.98 ms; inversion time = 400 ms; flip angle = 11°; voxel size 1.0 × 1.0 × 1.2 mm; for full details see http://adni.loni.usc.edu/methods/mri-analysis/mri-acquisition/). Structural T1 images were segmented into grey matter, white matter and cerebrospinal fluid (CSF) using Statistical Parametric Mapping software (SPM8; Wellcome Trust Centre for Neuroimaging, London, UK) to allow correction of the ^1^H-MRS data for partial volume CSF contamination. ^1^H-MRS spectra (PRESS–Point RESolved Spectroscopy; TE = 30 ms; TR = 3000 ms; 96 averages) were acquired in the left hippocampus (voxel dimensions: 20 × 20 × 15 mm (right–left, anterior–posterior, superior–inferior); see Supplementary Fig. 1). We employed the standard GE probe (proton brain examination) sequence, which uses a standardised chemically selective suppression (CHESS) water suppression routine. Unsuppressed water reference spectra (16 averages) were also acquired as part of the standard acquisition. Shimming and water suppression were optimised, with auto-prescan performed twice before each scan.

LC-model 6.3-I0 [46] was used to estimate glutamate levels. Following visual inspection of spectra quality, metabolite analyses were restricted to spectra with linewidths at full-width at half-maximum ≤0.1 ppm, Cramér–Rao lower bounds ≤20% and signal to noise ratio ≥5. Model metabolites and concentrations used in the basis set are fully detailed in the LC-Model manual (http://s-provencher.com/lcmodel.shtml). An in-house script was used to identify the relative distribution of white matter, grey matter and cerebrospinal fluid in the voxel. Metabolite values were corrected for the CSF content of the voxel using the formula Mcorr = M × (WM + 1.28 GM + 1.55 CSF)/(WM + GM), where M is the uncorrected metabolite value, and WM, GM and CSF are the white matter, grey matter and cerebrospinal fluid fractions of the voxel, respectively [47]. The ^1^H-MRS data are a sub-set of a larger sample recently reported [48], but the PET and the integration of the PET and ^1^H-MRS data have not been previously reported. The voxel tissue content and imaging quality control variables are summarised in Supplementary Table 9.

### Statistical analysis

All statistical analyses were performed using R version 3.3.2 [49]. Significance was set at *p* < 0.05 (two tailed). Baseline clinico-demographic variables were compared using independent *t* tests or ANOVA for the continuous data, and the chi-square test for categorical variables.

To test our first hypothesis that there was an inverse relationship between glutamate and dopamine in the high-risk sample as a whole, a linear regression analysis was performed with whole striatal dopamine synthesis capacity as the dependent variable, and glutamate concentrations as the predictor. We investigated whether this relationship was altered following the addition of potentially confounding variables (age, sex and ethnicity) to the model, given possible effects of age [50], sex [51, 52] and ethnicity [53], on the imaging measures.

To test our second hypothesis that dopamine synthesis capacity at baseline predicted worsening of symptoms, we performed linear regressions, analysing both the relationship between dopamine synthesis capacity and percentage change in symptoms. Given possible effects of age [50], sex [51, 52] and ethnicity [53], on the imaging measures, we conducted secondary exploratory analyses that adjusted for these co-variates.

In addition, we conducted exploratory analyses to determine if dopamine synthesis capacity predicted persistent functional impairment. We also investigated whether baseline dopamine levels were different from controls using independent *t* tests and ANCOVA.

## Results

Fifty-one patients and 19 controls participated in the study. Forty-seven patients received both PET and MRI scans (one received only an MRI scan and three received only a PET scan), and all controls received both PET and MRI scans. Time between scans was similar for patients (median 17.7 weeks, IQR 13.4–33.3) and controls (median 11.6 weeks, 4.3–23.3) (Mann–Whitney U Test, *p* = 0.82). There were no significant differences between patient and control groups in terms of age, gender, ethnicity or estimated premorbid IQ (see Table 1). Of the 51 patients, 35 were followed-up clinically, while 16 were lost to follow-up. There were no significant differences in clinico-demographic variables between the subjects with follow-up and those lost to follow-up (Table 2).

**Table 1:** Clinico-demographic details of study participants

	Controls	Patients	*P*
*n*	19	51	
Age	25.1(4.3)	23.0 (4.0)	0.06
% male	47	57	0.66^a^
% white ethnicity	79	73	0.81^a^
PANSS-positive	n/a	14.6 (4.8)	–
PANSS-negative	n/a	12.9 (4.7)	–
PANSS general	n/a	31.5 (9.7)	–
PANSS total	n/a	59.1 (16.9)	–
CAARMS-positive	n/a	10.4 (4.4)	–
CAARMS total	n/a	42.3 (20.9)	–
GAF	92.7 (5.7)	56.4 (9.6)	<0.001
Premorbid IQ estimate	102.0 (12.7)	105.1 (13.7)	0.41
Hippocampal glutamate concentration	8.06 (1.09)	8.27 (1.43)	0.57
Striatal K_i_^cer^	0.0128 (0.0011)	0.0126 (0.0010)	0.53

*PANSS*  Positive and Negative Syndrome Scale, *CAARMS*   comprehensive assessment of the at-risk mental state, *GAF*   general assessment of functioning, *IQ*   intelligence quotient estimated from national adult reading test

Values are mean (SD). *P-* values refer to control–patient comparison. *P*-values refer to *t* test unless otherwise indicated

^a^Chi-square test

**Table 2:** Clinico-demographic details of patients with and without clinical follow-up

	Sample with follow-up	Lost to follow-up	*P*
*n*	35	16	
Age	23.1 (3.9)	22.7 (4.5)	0.75
% male	60	50	0.72^a^
% white ethnicity	71	75	0.99^a^
PANSS-positive	14.1 (4.4)	15.8 (5.5)	0.25
PANSS-negative	12.2 (4.5)	12.3 (5.2)	0.52
PANSS general	31.9 (9.9)	30.7 (9.5)	0.70
PANSS total	59.3 (16.3)	58.7 (18.8)	0.91
CAARMS-positive	10.8 (4.4)	9.36 (4.31)	0.30
CAARMS total	43.4 (20.9)	42.4 (23.1)	0.89
GAF	55.0 (9.5)	59.4 (9.6)	0.14
Premorbid IQ estimate	107.0 (10.7)	101.1 (18.2)	0.16
Hippocampal glutamate concentration	8.26 (1.5)	8.29 (1.4)	0.95
Striatal K_i_^cer^	0.0127 (0.001)	0.0125 (0.001)	0.50

*PANSS*   Positive and Negative Syndrome Scale, *CAARMS*   comprehensive assessment of the at-risk mental state, *GAF*   general assessment of functioning, *IQ*   intelligence quotient estimated from national adult reading test

Values are mean (SD) or % in case of sex and ethnicity. *P*-values refer to *t* test unless otherwise indicated

^a^Chi-square test

### Imaging results in the total clinical high-risk sample relative to controls

**Fig. 1:**
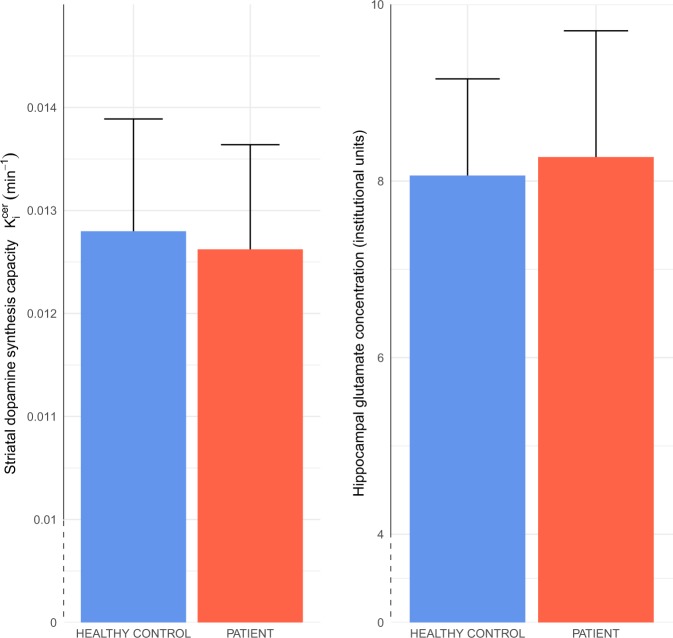
Mean (SD) striatal dopamine synthesis capacity and hippocampal glutamate concentrations are not significantly different between patients and controls (*p* = 0.55 and *p* = 0.52, respectively)

There were no significant differences between the total patient and control groups in terms of dopamine synthesis capacity (*t* = 0.63, df = 67, *p* = 0.53) or hippocampal glutamate concentrations (*t* = 0.57, df = 65, *p* = 0.52; see Fig. 1, Supplementary Fig. 2), and this remained the case after controlling for the influence of age, gender and ethnicity (group effect on dopamine synthesis capacity: *F* = 0.39, df = 64, *p* = 0.54; and on glutamate concentrations: *F* = 0.39, df = 62, *p* = 0.54). There were also no significant differences in dopamine synthesis capacity between groups for any of the striatal subdivisions (see Supplementary Information, Supplementary Tables 1, 2).

### Relationship between striatal dopamine synthesis capacity and hippocampal glutamate levels

**Fig. 2:**
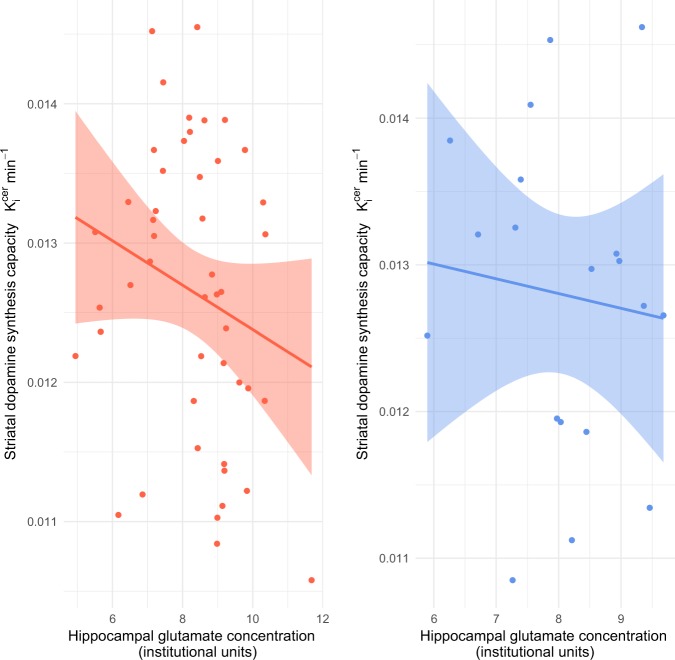
Hippocampal glutamate concentration is not significantly related to striatal dopamine synthesis capacity in patients (red; *p* = 0.13) or controls (blue, *p* = 0.68)

Figure 2 shows the relationship between hippocampal glutamate concentrations and striatal dopamine synthesis capacity in patients and controls. The bivariate regression showed that there was no significant association between hippocampal glutamate concentrations and striatal dopamine synthesis capacity in patients (beta = −1.60 × 10^−4^, SE = 1.05 × 10^−4^, *R*^2^ = 0.05, *p* = 0.13), including in the adjusted model (when age, gender and ethnicity were added to the model (beta = −1.92 × 10^−4^, SE = 1.14 × 10^−4^, *p* = 0.10)), or in controls in either the bivariate (beta = −1.01 × 10^−4^, SE = 2.40 × 10^−4^, *R*^2^ = 0.01, *p* = 0.68) or adjusted (beta = 5.92 × 10^−4^, SE = 2.85  × 10^−4^, *p* = 0.98) models. No significant relationships were seen for any of the striatal subdivisions either, in either patients or controls (see Supplementary Information, Supplementary Tables 5–8).

### Relationship between dopamine synthesis capacity and subsequent worsening of symptoms

**Fig. 3:**
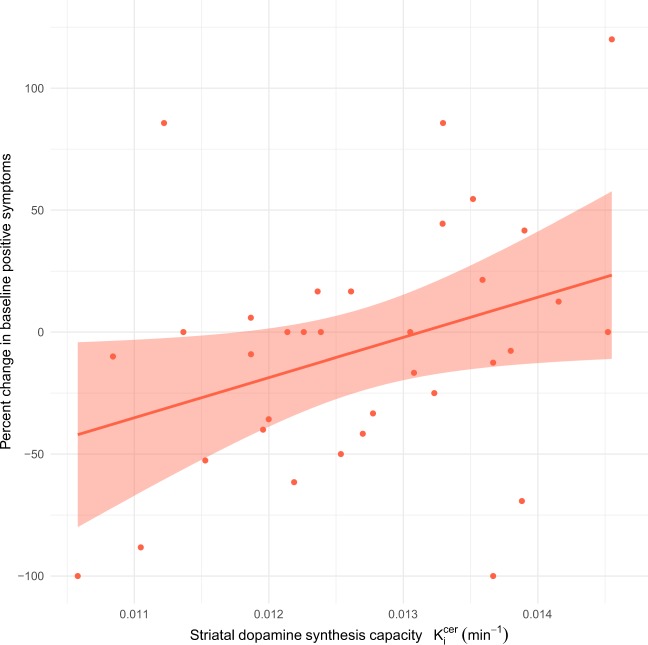
Striatal dopamine synthesis capacity at baseline is directly associated with subsequent increase (worsening) of psychotic (positive) symptoms (*r* = 0.35, *p* < 0.05) in people at clinical risk of psychosis

Dopamine synthesis capacity at baseline was associated with subsequent worsening of psychotic (positive) symptoms (beta = 1.6 × 10^4^, SE = 7.9 × 10^3^, *R*^2^ = 0.12, df = 32, *p* < 0.05) (see Fig. 3), and this remained significant when age, gender and ethnicity were added to the model (beta = 1.9 × 10^4^, SE = 8.5 × 10^3^, df = 29, *p* < 0.05). The relationship was significant for the associative subdivision, but not sensorimotor or limbic subdivisions (see Supplementary Information, Supplementary Tables 3, 4). The relationship was similar when the total symptom rather than the positive symptom scores were examined, in both unadjusted (beta = 4.5 × 10^4^, SE = 1.8 × 10^4^, df = 32, *p* < 0.05) and adjusted models (beta = 4.8 × 10^4^, SE = 2.0 × 10^4^, df = 29, *p* < 0.05).

### Transition to a psychotic disorder

Ten individuals (19.6% of the total high-risk sample) developed a psychotic disorder during the follow-up period after the PET and MRS scans. There was no significant difference in baseline dopamine synthesis capacity between the transition (mean (SD): 0.0122 (0.001)) and non-transition (mean (SD): 0.0127 (0.001)) sub-groups (*t* = 1.3, df = 48, *p* = 0.28).

## Discussion

Our first main finding is that in people at clinical high risk for psychosis, striatal dopamine synthesis capacity predicted the worsening of psychotic symptoms. This adds to evidence that dopamine synthesis and release capacity are positively correlated with psychotic symptom severity [54–56], and treatment response [57] in patients with a psychotic disorder. Although the relationship was strongest for the associative subdivision, specificity to this region remains to be established given the high degree of collinearity between dopamine measures for all three subdivisions and that we did not test for an interaction. However, there was no significant difference between patients who later developed a psychotic disorder relative to those who did not, contrary to our previous findings [32]. This could be due to the fact that the difference between receiving a diagnosis of a psychotic disorder or not can come down to small differences in duration or severity of symptoms, for example, the difference between 7 days versus 6 days of psychotic symptoms [34]. It has been argued that these differences are unlikely to be clinically meaningful, and that a dimensional approach to psychosis may be more appropriate [58]. This is also consistent with evidence that there is a dimensional relationship between dopamine dysregulation and the induction of psychotic symptoms in healthy volunteers as well [59–61]. Factors other than symptom levels may also influence the diagnostic process, such as the patient’s coping skills and level of functioning. It should also be noted that some patients in the non-transition group showed a greater worsening of symptoms than patients in the transition group (see Supplementary Fig. 3), and that some individuals develop a psychotic disorder up to 10 years after [62], indicating that further follow-up is required to determine if there are any transitions in the non-transition group. Notwithstanding this, taken together, our findings that there was a relationship between dopaminergic dysfunction and worsening of symptoms, but not transition to psychosis, indicate that dopamine dysfunction is more strongly linked to the development of symptoms than a diagnosis of a psychotic disorder. These findings add to other evidence that alterations in subcortical dopamine function in subjects with mental health problems may be more related to psychotic symptoms than to diagnostic categories per se [26, 54]. For example, patients with psychotic bipolar disorder show a similar elevation of striatal dopamine synthesis capacity to patients with a schizophreniform psychosis [54].

Our finding that there was no significant relationship between hippocampal glutamate levels and striatal dopamine synthesis capacity contrasts with our previous finding in subjects at clinical high risk of psychosis, which found a negative relationship with *r* = 0.54 [21]. It is possible that our failure to detect a relationship in the current study is due to a type II error. However, the patient sample in this study (*n* = 51) was much larger than in the previous study (*n* = 14), and had >80% power to detect the anticipated moderate or larger (*r* > 0.4) relationship between dopamine and glutamate indices. Thus, our study was well powered to detect the anticipated effect size, although it is possible that there is a smaller effect. It should be recognised that the MRS glutamate signal at 3 T is a composite of intra and extrasynaptic glutamate, and glutamine [3, 63]. Thus, we cannot exclude the possibility that alterations in synaptic glutamate levels are masked by other components of the signal, or indeed, that there are alterations in glutamate receptor levels. Notwithstanding these caveats, our findings are not consistent with the model that increased hippocampal glutamate levels dysregulates striatal dopamine function. They do not, however, rule out the alternative hypothesis suggested by preclinical models and clinical findings of hippocampal overactivity in psychosis [8, 12, 13, 18–20], that it is disinhibition of glutamate output neurons, and not altered glutamate drive in the hippocampus, that leads to subcortical dopaminergic dysregulation. This disinhibition could occur secondary to reduced GABAergic interneuron function, or other mechanisms affecting glutamatergic neuronal excitability that occur without concomitant measurable differences in glutamate concentrations in the hippocampus. In this case, one would not predict a relationship between hippocampal glutamate levels and striatal dopaminergic function, as it would be glutamate levels at the site of the projections’ termination (i.e., the striatum) that would show an association with dopamine function. A recent study has reported an inverse relationship between glutamate concentration in the anterior cingulate cortex and striatal dopamine synthesis capacity in patients with first-episode psychosis [64], and this has been seen also in healthy controls along with a direct association between striatal glutmate levels and striatal dopamine synthesis capacity [65]. Unfortunately, we did not measure glutamate concentration in the striatum or anterior cingulate cortex in this study. Further studies are warranted to test the relationship between striatal dopamine function and glutamate concentration in other brain regions in high-risk subjects.

However, an alternative hypothesis is that it is disinhibition of hippocampal glutamatergic output neurons, as opposed to glutamatergic drive in the hippocampus, that leads to striatal dopaminergic dysregulation [8],

We did not find a difference in striatal dopaminergic function between clinical high-risk subjects overall and controls, in contrast to previous findings [28, 29, 66]. This difference could reflect changes in the population referred to early detection services over time, with evidence indicating that subjects are referred earlier in the at risk period in more recent cohorts compared with earlier cohorts [67]. This is consistent with the transition rate in the present sample, which was ~19%, compared with ~35% in earlier samples [28, 32]. Transition rates similar to those in our current sample have also been reported in more recent cohorts from clinical studies around the world [2, 62], indicating that our current sample is likely to be representative of subjects currently referred to at risk services. Nevertheless, the lower transition rate and evidence that there may be transitions up to 10 years after presentation [62], indicates that the current finding of no difference in dopamine synthesis capacity should be considered as preliminary pending long-term follow-up of our current sample. The potential lack of generalisability to cohorts, where transition rates are greater, is a limitation that pertains to all the negative findings reported. We also did not detect a significant difference in hippocampal glutamatergic function between the high-risk subjects and controls, in contrast to our previous findings in a larger study that included the current cohort [48], although in agreement with previous studies in smaller samples that also did not detect significant differences [21, 68]. Thus, the difference between the Bossong et al. finding [48] and our current results may reflect the lower power in the current sample.

### Methodological considerations

A number of subjects were lost to follow-up, which could introduce bias into the outcomes. However, the clinical and demographic characteristics of these subjects were not significantly different from those in the other groups, indicating this unlikely to be a major bias. It should be recognised that some non-transition subjects might subsequently develop a psychotic disorder. However, as the peak period for transition to psychosis is within the first year of follow-up [2], it is likely that we have identified the majority of transitions. Although it was not significant, there was a trend for the controls to be older than the patients. However, including age as a covariate in analyses did not have a major effect on findings.

### Implications

Our finding that dopamine synthesis capacity predicted the worsening of psychotic symptoms but was not linked to transition suggests that other factors are involved in the diagnosis of psychotic disorder. One interpretation could be that dopamine dysfunction underlies the development of psychotic symptoms, but whether these have a functional impact depends on additional factors, such as the coping skills, and psychological response of the individual, and their social support, consistent with psychosocio-biological models of psychosis [69–71]. Another possibility is that in the at-risk period, small differences in dopamine drive short-term psychotic-like experiences, but whether these become long-lasting and more severe depends on the development of further dopamine dysregulation. These possibilities are not mutually exclusive and a combination of both is possible [69].

Our finding that hippocampal glutamate is not linked to striatal dopamine dysfunction does not support the hypothesis that elevated hippocampal glutamatergic drive is driving striatal dopamine dysfunction, but is consistent with models that disinhibition of glutamatergic projections could drive striatal dopamine dysregulation. This predicts increased glutamate levels in targets of glutamatergic projections from the hippocampus, including the striatum. Elevated glutamate levels have been reported in the striatum in people at risk of psychosis, and linked to the transition to psychosis [72, 73]. Unfortunately, we did not measure striatal glutamate levels due to time constraints. New methods to index inhibitory regulation of hippocampal projection neurons, and MRS studies involving the targets of hippocampal projection neurons, are needed to test this further.

## Conclusions

Striatal dopamine synthesis capacity predicts worsening of psychotic-like symptoms, but is not strongly related to transition to psychosis or hippocampal glutamate levels, indicating a role for dopamine in the development of symptoms but that other factors contribute to the transition to a psychotic disorder.

## Funding and disclosure

This study was funded by Medical Research Council-UK, and Wellcome Trust grants and the National Institute for Health Research (NIHR) Biomedical Research Centre at South London and Maudsley NHS Foundation Trust and King’s College London. Dr Howes has received investigator-initiated research funding from and/or participated in advisory/speaker meetings organised by Angellini, Astra-Zeneca, Autifony, Biogen, BMS, Eli Lilly, Heptares, Jansenn, Lundbeck, Lyden-Delta, Otsuka, Servier, Sunovion, Rand and Roche. Neither Dr Howes nor his family have been employed by or have holdings/a financial stake in any pharmaceutical company. The other authors have no relevant conflicts of interest.

## Supplementary information

Supplementary material

## References

[1] Yung AR, Phillips LJ, Yuen HP, Francey SM, McFarlane CA, Hallgren M (2003). Psychosis prediction: 12-month follow up of a high-risk (‘prodromal’) group. Schizophr Res..

[2] Fusar-Poli P, Bonoldi I, Yung AR, Borgwardt S, Kempton MJ, Valmaggia L (2012). Predicting psychosis: meta-analysis of transition outcomes in individuals at high clinical risk. Arch Gen Psychiatry.

[3] Howes O, McCutcheon R, Stone J (2015). Glutamate and dopamine in schizophrenia: an update for the 21 st century. J Psychopharmacol..

[4] Millan MJ, Andrieux A, Bartzokis G, Cadenhead K, Dazzan P, Fusar-Poli P (2016). Altering the course of schizophrenia: progress and perspectives. Nat Rev Drug Discov..

[5] Moghaddam B, Javitt D (2012). From revolution to evolution: the glutamate hypothesis of schizophrenia and its implication for treatment. Neuropsychopharmacol.

[6] Coyle J (2006). Glutamate and schizophrenia: beyond the dopamine hypothesis. Cell Mol Neurobiol..

[7] Goff DC, Coyle JT (2001). The emerging role of glutamate in the pathophysiology and treatment of schizophrenia. Am J Psychiatry..

[8] Grace AA, Gomes FV. The circuitry of dopamine system regulation and its disruption in schizophrenia: insights into treatment and prevention. Schizophr Bull. 2018;45:1–10. 10.1093/schbul/sbx199 PMC6293217 29385549

[9] McCutcheon RA, Abi-dargham A, Howes OD (2019). Schizophrenia, dopamine and the striatum: from biology to symptoms. Trends Neurosci.

[10] Grace A (2012). Dopamine system dysregulation by the hippocampus: Implications for the pathophysiology and treatment of schizophrenia. Neuropharmacology..

[11] Grace AA (2016). Dysregulation of the dopamine system in the pathophysiology of schizophrenia and depression. Nat Rev Neurosci..

[12] Lodge DJ, Grace AA (2007). Aberrant hippocampal activity underlies the dopamine dysregulation in an animal model of schizophrenia. J Neurosci.

[13] Schobel Sa, Chaudhury NH, Khan Ua, Paniagua B, Styner Ma, Asllani I (2013). Imaging patients with psychosis and a mouse model establishes a spreading pattern of hippocampal dysfunction and implicates glutamate as a driver. Neuron..

[14] Pantelis C, Velakoulis D, McGorry PD, Wood SJ, Suckling J, Phillips LJ (2003). Neuroanatomical abnormalities before and after onset of psychosis: a cross-sectional and longitudinal MRI comparison. Lancet..

[15] Walter A, Studerus E, Smieskova R, Kuster P, Aston J, Lang UE (2012). Hippocampal volume in subjects at high risk of psychosis: a longitudinal MRI study. Schizophr Res..

[16] Stan AD, Ghose S, Zhao C, Hulsey K, Mihalakos P, Yanagi M (2015). Magnetic resonance spectroscopy and tissue protein concentrations together suggest lower glutamate signaling in dentate gyrus in schizophrenia. Mol Psychiatry..

[17] Brugger SP, Howes OD (2017). Heterogeneity and homogeneity of regional brain structure in schizophrenia. JAMA Psychiatry..

[18] Heckers S, Konradi C (2010). Hippocampal pathology in schizophrenia. Curr Top Behav Neurosci..

[19] Allen P, Chaddock CA, Egerton A, Howes OD, Bonoldi I, Zelaya F (2016). Resting hyperperfusion of the hippocampus, midbrain, and basal ganglia in people at high risk for psychosis. Am J Psychiatry..

[20] Allen P, Azis M, Modinos G, Bossong MG, Bonoldi I, Samson C (2018). Increased resting hippocampal and basal ganglia perfusion in people at ultra high risk for psychosis: replication in a second cohort. Schizophr Bull.

[21] Stone JM, Howes OD, Egerton A, Kambeitz J, Allen P, Lythgoe DJ (2010). Altered relationship between hippocampal glutamate levels and striatal dopamine function in subjects at ultra high risk of psychosis. Biol Psychiatry..

[22] Howes OD, Kambeitz J, Stahl D, Slifstein M, Abi-Dargham A, Kapur S (2012). The nature of dopamine dysfunction in schizophrenia and what this means for treatment. Arch Gen Psychiatry..

[23] McCutcheon R, Beck K, Jauhar S, Howes OD (2018). Defining the locus of dopaminergic dysfunction in schizophrenia: a meta-analysis and test of the mesolimbic hypothesis. Schizophr Bull.

[24] Howes OD, Williams M, Ibrahim K, Leung G, Egerton A, McGuire PK (2013). Midbrain dopamine function in schizophrenia and depression: a post-mortem and positron emission tomographic imaging study. Brain..

[25] Abi-Dargham a, Gil R, Krystal J, Baldwin RM, Seibyl JP, Bowers M (1998). Increased striatal dopamine transmission in schizophrenia: confirmation in a second cohort. Am J Psychiatry..

[26] Reith J, Benkelfat C, Sherwin A, Yasuhara Y, Kuwabara H, Andermann F (1994). Elevated dopa decarboxylase activity in living brain of patients with psychosis. Proc Natl Acad Sci USA..

[27] Laruelle M, Abi-dargham A, Van Dyck CH, Gil R, Souza CDD, Erdos J (1996). Single photon emission computerized tomography imaging of schizophrenic subjects. Proc Natl Acad Sci USA..

[28] Howes OD, Montgomery AJ, Asselin MC, Murray RM, Valli I, Tabraham P (2009). Elevated striatal dopamine function linked to prodromal signs of schizophrenia. Arch Gen Psychiatry..

[29] Egerton A, Chaddock Ca, Winton-Brown TT, Bloomfield MaP, Bhattacharyya S, Allen P (2013). Presynaptic striatal dopamine dysfunction in people at ultra-high risk for psychosis: findings in a second cohort. Biol Psychiatry..

[30] Mizrahi R, Kenk M, Suridjan I, Boileau I, George TP, McKenzie K (2013). Stress-induced dopamine response in subjects at clinical high risk for schizophrenia with and without concurrent cannabis use. Neuropsychopharmacology..

[31] Howes OD, Shotbolt P, Bloomfield M, Daalman K, Demjaha A, Diederen KMJ (2013). Dopaminergic function in the psychosis spectrum: an [18F]-DOPA imaging study in healthy individuals with auditory hallucinations. Schizophr Bull.

[32] Howes O, Bose S, Turkheimer FE, Valli I, Egerton A, Valmaggia L (2011). Dopamine synthesis capacity before onset of psychosis: a prospective -DOPA PET imaging study. Am J Psychiatry..

[33] Howes O, Bose S, Turkheimer F, Valli I, Egerton A, Stahl D (2011). Progressive increase in striatal dopamine synthesis capacity as patients develop psychosis: a PET study. Mol Psychiatry..

[34] Yung AR, Yuen HP, McGorry PD, Phillips LJ, Kelly D, Dell’Olio M (2005). Mapping the onset of psychosis: the comprehensive assessment of at-risk mental states. Aust N Z J Psychiatry..

[35] Spitzer R, Williams J, Gibbon M (1994). Structured clinical interview for DSM-IV.

[36] Jones SH, Thornicroft G, Coffey M, Dunn G (1995). A brief mental health outcome scale-reliability and validity of the global assessment of functioning (GAF). Br J Psychiatry..

[37] Nelson HE (1982). The national adult reading test (NART): test manual. Wind UK NFER-Nelson..

[38] Kay SR, Flszbein A, Opfer LA (1987). The positive and negative syndrome scale (PANSS) for schizophrenia. Schizophr Bull..

[39] Obermeier M, Mayr A, Schennach-wolff R, Seemu F (2010). Should the PANSS be rescaled?. Schizophr Bull.

[40] Bloomfield MA, Pepper F, Egerton A, Demjaha A, Tomasi G, Mouchlianitis E, et al. Dopamine function in cigarette smokers: an [(18)F]-DOPA PET study. Neuropsychopharmacology. 2014;39:2397. 10.1038/npp.2014.87 PMC4138749 24718373

[41] Montgomery AJ, Thielemans K, Mehta MA, Turkheimer F, Mustafovic S, Grasby PM (2006). Correction of head movement on PET studies: comparison of methods. J Nucl Med.

[42] Jauhar S, Veronese M, Rogdaki M, Bloomfield M, Natesan S, Turkheimer F (2017). Regulation of dopaminergic function: an [18F]-DOPA PET apomorphine challenge study in humans. Transl Psychiatry.

[43] Martinez D, Slifstein M, Broft A, Mawlawi O, Hwang D, Huang Y (2003). Imaging human mesolimbic dopamine transmission with positron emission tomography. Part II: amphetamine-induced dopamine release in the functional subdivisions of the striatum. J Cereb Blood Flow Metab..

[44] Patlak CS, Blasberg RG (1985). Graphical evaluation of blood-to-brain transfer constants from multiple-time uptake data. Generalizations. J Cereb Blood Flow Metab..

[45] Egerton A, Demjaha A, McGuire P, Mehta MA, Howes OD (2010). The test-retest reliability of 18F-DOPA PET in assessing striatal and extrastriatal presynaptic dopaminergic function. Neuroimage..

[46] LCModel’s home page. http://s-provencher.com/lcmodel.shtml.

[47] Gasparovic C, Song T, Devier D, Bockholt HJ, Caprihan A, Mullins PG (2006). Use of tissue water as a concentration reference for proton spectroscopic imaging. Magn Reson Med..

[48] Bossong MG, Antoniades M, Azis M, Samson C, Quinn B, Bonoldi I, et al. Elevated hippocampal glutamate levels associated with adverse outcomes in people at clinical high risk for psychosis. JAMA Psychiatry. 2018;76:199–207. 10.1001/jamapsychiatry.2018.3252 PMC6440239 30427993

[49] R: The R Project for Statistical Computing. https://www.r-project.org/.

[50] Braskie MN, Wilcox CE, Landau SM, O’Neil JP, Baker SL, Madison CM (2008). Relationship of striatal dopamine synthesis capacity to age and cognition. J Neurosci..

[51] Laakso A, Vilkman H, Bergman JÖ, Haaparanta M, Solin O, Syvälahti E (2002). Sex differences in striatal presynaptic dopamine synthesis capacity in healthy subjects. Biol Psychiatry..

[52] Tayoshi S, Sumitani S, Taniguchi K, Shibuya-Tayoshi S, Numata S, Iga JI (2009). Metabolite changes and gender differences in schizophrenia using 3-Tesla proton magnetic resonance spectroscopy (1H-MRS). Schizophr Res..

[53] Egerton A, Howes OD, Houle S, McKenzie K, Valmaggia LR, Bagby MR (2017). Elevated striatal dopamine function in immigrants and their children: a risk mechanism for psychosis. Schizophr Bull..

[54] Jauhar S, Nour MM, Veronese M, Rogdaki M, Bonoldi I, Azis M (2017). A test of the transdiagnostic dopamine hypothesis of psychosis using positron emission tomographic imaging in bipolar affective disorder and schizophrenia. JAMA Psychiatry..

[55] Laruelle M, Abi-Dargham A, Gil R, Kegeles L, Innis R (1999). Increased dopamine transmission in schizophrenia: relationship to illness phases. Biol Psychiatry..

[56] Thompson JL, Urban N, Slifstein M, Xu X, Kegeles LS, Girgis RR (2013). Striatal dopamine release in schizophrenia comorbid with substance dependence. Mol Psychiatry..

[57] Jauhar S, Veronese M, Nour MM, Rogdaki M, Hathway P, Turkheimer FE, et al. Determinants of treatment response in first-episode psychosis: an 18F-DOPA PET study. Mol Psychiatry. 2018. (advance online publication, 20 April 2018). 10.1038/s41380-018-0042-4. 10.1038/s41380-018-0042-4 PMC6331038 29679071

[58] van Os J, Linscott RJ, Myin-Germeys I, Delespaul P, Krabbendam L (2009). A systematic review and meta-analysis of the psychosis continuum: evidence for a psychosis proneness-persistence-impairment model of psychotic disorder. Psychol Med..

[59] Nour MM, Dahoun T, Schwartenbeck P, Adams RA, FitzGerald THB, Coello C (2018). Dopaminergic basis for signaling belief updates, but not surprise, and the link to paranoia. Proc Natl Acad Sci.

[60] Woodward ND, Cowan RL, Park S, Ansari MS, Baldwin RM, Li R (2011). Correlation of individual differences in schizotypal personality traits with amphetamine-induced dopamine release in striatal and extrastriatal brain regions. Am J Psychiatry..

[61] Soliman A, O’Driscoll Ga, Pruessner J, Joober R, Ditto B, Streicker E (2011). Limbic response to psychosocial stress in schizotypy: a functional magnetic resonance imaging study. Schizophr Res..

[62] Nelson B, Yuen HP, Wood SJ, Lin A, Spiliotacopoulos D, Bruxner A (2013). Long-term follow-up of a group at ultra high risk (“Prodromal”) for psychosis. JAMA Psychiatry..

[63] Poels EMP, Kegeles LS, Kantrowitz JT, Slifstein M, Javitt DC, Lieberman JA (2014). Imaging glutamate in schizophrenia: review of findings and implications for drug discovery. Mol Psychiatry..

[64] Jauhar S, McCutcheon R, Borgan F, Veronese M, Nour MM, Pepper F (2018). The relationship between cortical glutamate and striatal dopamine function in first episode psychosis: a multi-modal PET and MRS imaging study. Lancet Psychiatry..

[65] Gleich T, Deserno L, Lorenz RC, Boehme R, Pankow A, Buchert R (2015). Prefrontal and striatal glutamate differently relate to striatal dopamine: potential regulatory mechanisms of striatal presynaptic dopamine function?. J Neurosci..

[66] Mizrahi R, Addington J, Rusjan PM, Suridjan I, Ng A, Boileau I (2012). Increased stress-induced dopamine release in psychosis. Biol Psychiatry..

[67] Fusar-Poli P, Schultze-Lutter F, Cappucciati M, Rutigliano G, Bonoldi I, Stahl D (2016). The dark side of the moon: meta-analytical impact of recruitment strategies on risk enrichment in the clinical high risk state for psychosis. Schizophr Bull..

[68] Stone JM, Day F, Tsagaraki H, Valli I, McLean MA, Lythgoe DJ (2009). Glutamate dysfunction in people with prodromal symptoms of psychosis: relationship to gray matter volume. Biol Psychiatry..

[69] Howes O, Murray R (2014). Schizophrenia: an integrated sociodevelopmental-cognitive model. Lancet..

[70] Van Winkel R, Stefanis NC, Myin-Germeys I (2008). Psychosocial stress and psychosis. A review of the neurobiological mechanisms and the evidence for gene-stress interaction. Schizophr Bull..

[71] Howes OD, McCutcheon R, Owen MJ, Murray RM (2017). The role of genes, stress, and dopamine in the development of schizophrenia. Biol Psychiatry..

[72] de la Fuente-Sandoval C, León-Ortiz P, Favila R, Stephano S, Mamo D, Ramírez-Bermúdez J (2011). Higher levels of glutamate in the associative-striatum of subjects with prodromal symptoms of schizophrenia and patients with first-episode psychosis. Neuropsychopharmacology..

[73] de la Fuente-Sandoval C, Leon-Ortiz P, Azcárraga M, Favila R, Stephano S, Graff-Guerrero A (2013). Striatal glutamate and the conversion to psychosis: a prospective 1 H-MRS imaging study. Int J Neuropsychopharmacol..

