# Supplementary material for: Glutamatergic and dopaminergic function and the relationship to outcome in people at clinical high risk of psychosis: a multi-modal PET-magnetic resonance brain imaging study
Source: Neuropsychopharmacology. 2019 Oct 16;45(4):641–8. doi: 10.1038/s41386-019-0541-2 (PMC7021794; doi:10.1038/s41386-019-0541-2)
Supplement: Supplementary file 1 — Supplementary material [file 41386_2019_541_MOESM1_ESM.pdf]

Glutamatergic and dopaminergic function and the relationship to outcome in people at clinical high risk of psychosis: a multi-modal PET-magnetic resonance brain imaging study

Supplementary information

Supplementary results

Details of the 1H-MRS acquisition are shown in eFigure 1 and eTable 9.

Patient-control comparison of mean dopamine synthesis capacity and hippocampal glutamate concentration shown in eFigure 2

Percentage change in CAARMS positive symptoms between the transition and non-transition groups (efigure 3).

There were no significant differences in dopamine synthesis capacity between the total at risk patient group and the control group in any of the striatal sub-divisions (e-table 1), including after adjusting for age, gender and ethnicity (e-table 2).

Dopamine synthesis capacity was significantly directly correlated with symptom severity in the associative striatum, but not limbic or sensorimotor divisions (e-table 3), and this remained the case after adjusting for age, gender and ethnicity (e-table 4).

There were no significant relationships between hippocampal glutamate levels and dopamine synthesis capacity in any sub-division in patients (e-table 5), including after adjusting for age, gender and ethnicity (e-table 6), or in controls (e-table 7), including after adjusting for age, gender and ethnicity (e-table 8).

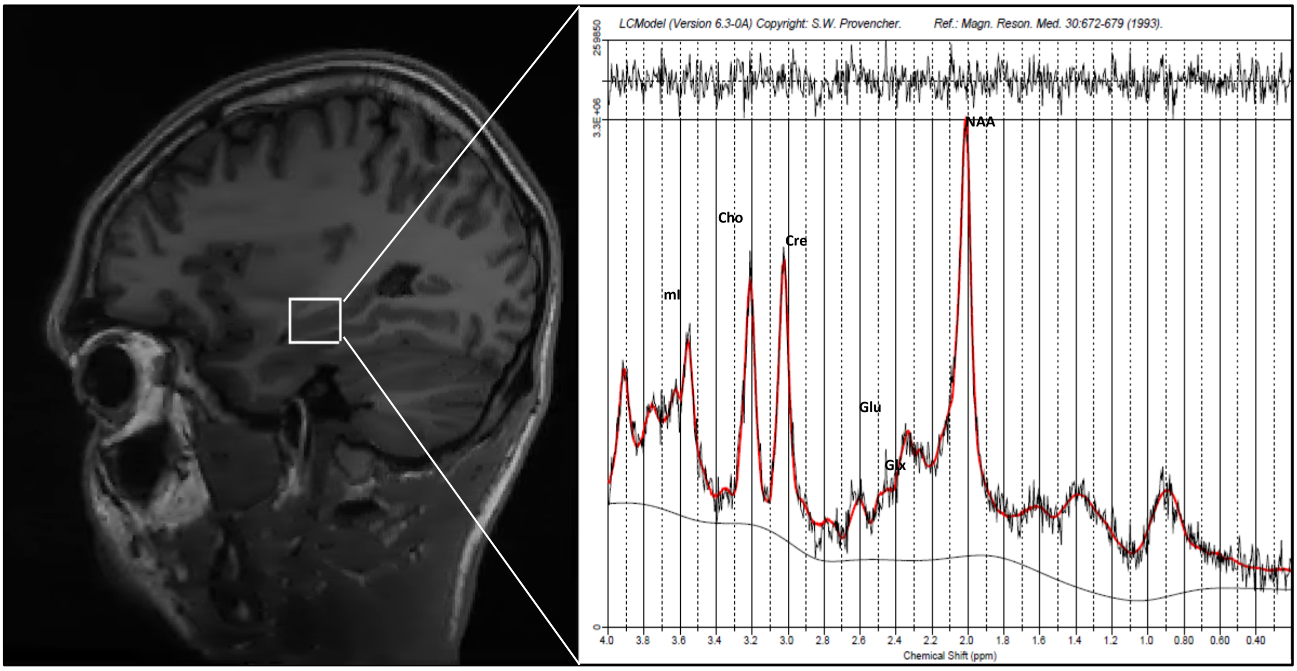

efigure 1. Positioning of 1H-MRS voxel in the left hippocampus and 1H-MRS spectrum obtained from the voxel (black line) and the overlay of the spectral fit (red line). All spectra were analyzed with LCModel version 6.3-0A (S.W. Provencher). Cho indicates choline; Cre, creatine; Glu, glutamate; Glx, combined measure of glutamine and glutamate; mI, myo-inositol; NAA, N-acetylasparate; and ppm, parts per million.

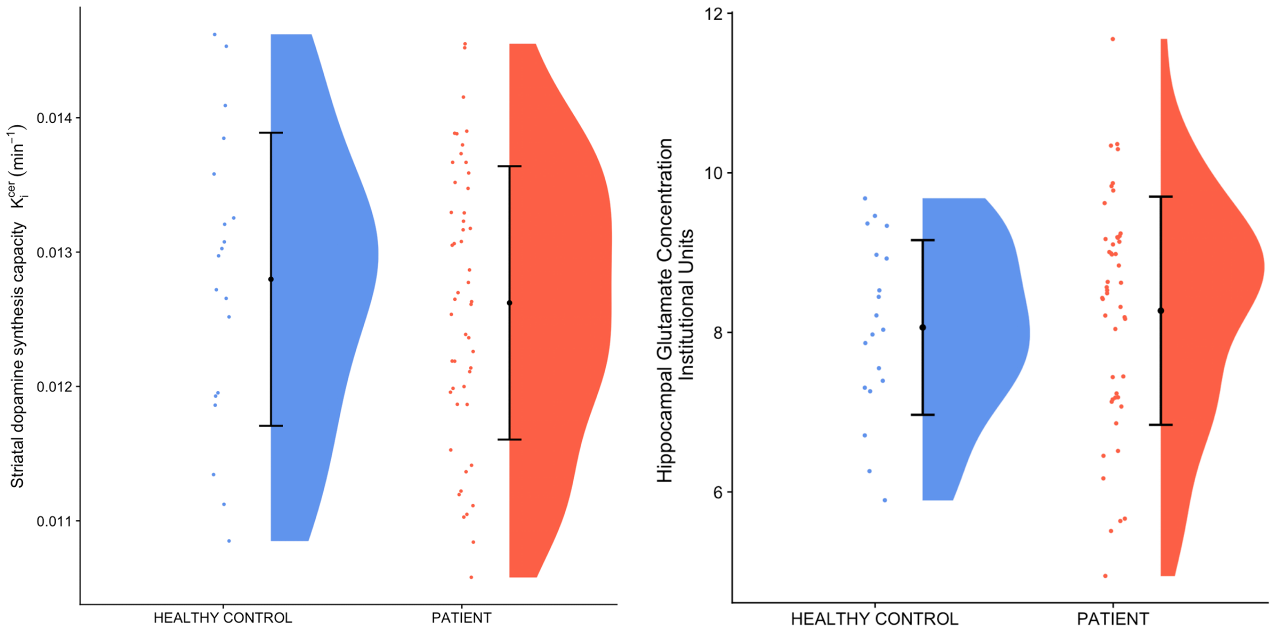

eFigure 2
Mean striatal dopamine synthesis capacity and hippocampal glutamate concentrations are not significantly different between patient and control groups (p=0.55 and p=0.52 respectively). Individual data points shown, with mean +/- SD in black, and kernel density plots to the right

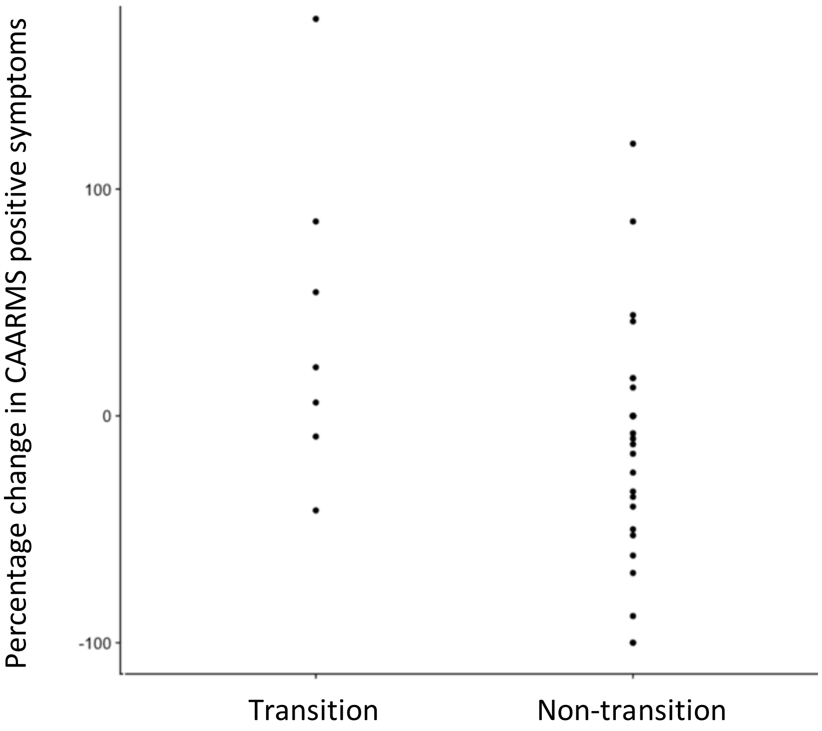

eFigure 3. Percentage change in CAARMS positive scores in individuals that did (transition group) and did not transition (non-transition group) to psychosis

Patient/Control differences:

	t	Degrees freedom	p
Limbic	1.18	67	0.24
Associative	0.61	67	0.55
Sensorimotor	0.39	67	0.70

e-table 1

	F	Degrees freedom	p
Limbic	1.36	64	0.25
Associative	0.36	64	0.55
Sensorimotor	0.16	64	0.69

e-table 2 (adjusted for age, gender and ethnicity)

Symptom -Dopamine Relationships – CAARMS symptoms

POSITIVE	t	Degrees freedom	p
Limbic	1.86	32	0.08
Associative	2.11	32	0.04
Sensorimotor	1.78	32	0.09
Whole	2.15	32	0.04
TOTAL			
Limbic	2.05	32	0.049
Associative	2.53	32	0.02
Sensorimotor	2.02	32	0.05
Whole	2.47	32	0.02

e-table 3

POSITIVE	F	Degrees freedom	p
Limbic	1.92	29	0.07
Associative	2.29	29	0.03
Sensorimotor	1.89	29	0.07
Whole	2.26	29	0.03
TOTAL			
Limbic	2.02	29	0.05
Associative	2.48	29	0.02
Sensorimotor	1.92	29	0.07
Whole	2.41	29	0.02

e-table 4 (adjusted for age, gender and ethnicity)

Dopamine-Glutamate relationships (patients)

	t	Degrees freedom	p
Limbic	-1.02	45	0.31
Associative	-1.54	45	0.13
Sensorimotor	-1.47	45	0.15

e-table 5

	t	Degrees freedom	p
Limbic	-1.11	42	0.28
Associative	-1.58	42	0.12
Sensorimotor	-1.90	42	0.06

e-table 6 (adjusted for age, gender and ethnicity)

Dopamine-Glutamate relationships (controls)

	t/F	Degrees freedom	p
Limbic	-1.02	17	0.32
Associative	-0.81	17	0.43
Sensorimotor	0.57	17	0.56

e-table 7

	t/F	Degrees freedom	p
Limbic	0.05	14	0.96
Associative	-0.19	14	0.85
Sensorimotor	0.41	14	0.69

e-table 8 (adjusted for age, gender and ethnicity)

1H-MRS voxel details

	**Controls**	**Patients**	**P**
**Voxel gray matter content**	63 (6)%	63 (7)%	0.94
**Voxel white Matter content**	34(6)%	33(8)%	0.72
**Voxel CSF Content**	2.7(1)%	3.5(2)	0.08
**SNR**	14.1(2.9)	13.4(2.8)	0.42
**FWHM**	8.8(2.5)ppm	8.5(1.9)ppm	0.66
**CRLB**	8.9(1.4)	9.6(2.1)	0.22

SNR – signal to noise ratio
FWHM-full width half maximum
CRLB – Cramer-Rao lower bounds

The quality of shimming can be estimated from the linewidth of the spectra. In this case mean linewidth is 8.57 ppm, indicating adequate shimming. All obtained spectra were included in the analysis.

Patient and control values compared using an independent sample t-test

e-table 9.
